# Hydrophilic melanin as a potential hair growth stimulant: A secondary observational study

**DOI:** 10.1111/jocd.16504

**Published:** 2024-07-29

**Authors:** S. V. Avetisyan, H. O. Koloyan, M. H. Paronyan, T. R. Petrosyan, A. S. Hovsepyan

**Affiliations:** ^1^ Scientific and Production Center “Armbiotechnology” SNPO NAS RA Yerevan Armenia; ^2^ Medical Institute, Yerevan Haybusak University Yerevan Armenia

To the Editor

Many manufacturers use melanins from different sources to produce cosmetics. Melanin inhibits the expression of inflammation‐associated enzymes, matrix metalloproteinases, and restores levels of cutaneous antioxidant enzymes.^1^ Study of hydrophilic bacterial melanin (BM) effects was initiated with experiments on plants. BM was obtained from the mutant strain of Bacillus Thuringiensis at the Institute of Biotechnology in Armenia. It was shown to be a potent stimulator of plant growth.^2^ BM has been tested in animal models to assess its effects on neuronal viability and regeneration. Topical injection of BM exhibited stimulatory action on axonal growth in experiments with injury of motor tracts and peripheral nerves,^3^ comanifested with better neuronal viability, neovascularization with extensive dilation of capillaries, suppressed inflammatory response and other effects (^4^). In our study the skin of animals was exposed to the pigment as well. Monitoring of the operated animals revealed stimulated hair growth in operated rats. To clarify this effect, we applied melanin solution (9 mg/mL for 10 consecutive days) to the larger cleaned lower back skin area (2 cm) in the experimental group rats (*n* = 12) and compared them to control animals with the same shaved skin area (*n* = 12). The skin of the study animals was observed every 3 days in order to register the hair regrowth pattern. One of the most commonly used hair growth assessment scale was offered by Matsuda et al, where the 0–4 scores are scaled the following way: no hair growth, less than 20%, 20%–39%, 40%–59%, 60%–79% and 80%–100% of hair growth.^5^ Another method used by many research groups is the self‐designed scale of hair regrowth (high hair density and full, thick fur (Level 4)); moderate hair density with no visible skin area (Level 3), low hair density, with the visualization of the skin (Level 2), uneven hair growth on the tested area, skin easily seen (Level 1).^6^ Significant hair growth was observed in all 12 experimental rats treated topically with melanin. The average growth in the study and control groups was measured on the 10th, 20th, and 30th days after the topical use of melanin solution (Table 1). The rat head has the shortest hair regrowth period of 4 weeks. The growth rate decreases to the lower back where the molt period is around 8 weeks. Microscopic analysis showed that BM affects the hair anagen phase, prolonging it and increasing anagen/telogen ratio. The skin examination in the experimental group showed expressed vasodilation in skin areas exposed to melanin. This effect was not observed in the control rats. Many hair growth stimulants possess vasodilatory action (e.g., Minoxidil). However, the future for the alopecia management and hair stimulant development is not limited to vasodilators or antiandrogenic agents. The regenerative medicine has suggested new approaches for the hair growth technology including the use of stem cells and growth factors.

**TABLE 1:** Hair growth results in experimental and control groups.

Days of assessment	Experimental group (*n*‐12)		Control group (*n*‐12)	
	Matsuda et al scoring	Self‐designed scale	Matsuda et al scoring	Self‐designed scale
0	No hair growth	–	No hair growth	–
10	Less than 20%	Level 2	Less than 20%	Level 1
20	40%–59%	Level 3	Less than 20%	Level 2
30	80%–100%	Level 4	20%–39%	Level 2

The above mentioned stimulation of axonal (neuritic) growth by the hydrophilic melanin is mediated by endogenous neurotrophic growth factors, which include NGF, BDNF, GDNF, and IGF‐1. The last factor is a confirmed regulator of hair growth cycle supporting the anagen stage and was shown to affect hair morphogenesis.

These preliminary data constitute the background to assess possible use of the water‐soluble and low‐cost BM as a proper topical therapeutic agent for inducing hair growth in a case control study. As a hair‐growth stimulant the BM acts with “double‐hit effect” including vasodilation and enhanced regeneration.

## AUTHOR CONTRIBUTIONS

All authors have read and approved the final manuscript. **P.T.R. and H.A.S** designed the research study. **P.T.R., H.A.S, A.S.V., K.H.O** performed the research. **A.S.V. and P.M.H** analyzed data. **P.T.R. and A.S.V.** wrote the paper.

## CONFLICT OF INTEREST STATEMENT

None declared. All authors have read and apprsoved the final version of the manuscript.

## ETHICS STATEMENT

The protocol was approved by the Institutional Animal Care and Ethics Committee of SNPO NAS RA in accordance with the European Communities Council Directive (86/609/EEC) on care and use of animals for experimental procedures (N8‐6/19, 21 March 2019). All applicable institutional and/or national guidelines for the care and use of animals were followed.

## Data Availability

Not Applicable.
